# The stromal-tumor amplifying STC1-Notch1 feedforward signal promotes the stemness of hepatocellular carcinoma

**DOI:** 10.1186/s12967-023-04085-8

**Published:** 2023-03-31

**Authors:** Shuya Bai, Yuchong Zhao, Wei Chen, Wang Peng, Yun Wang, Si Xiong, Aruna, Yanling Li, Yilei Yang, Shiru Chen, Bin Cheng, Ronghua Wang

**Affiliations:** 1 Department of Gastroenterology and Hepatology, Tongji Hospital, Tongji Medical College, Huazhong University of Science and Technology, 1095 Jiefang Avenue, Wuhan, 430030 China; 2 Department of Surgery, University of Pittsburgh School of Medicine, Pittsburgh, PA 15213 USA; 3 Department of Hepatobiliary and Pancreatic Surgery, The First Affiliated Hospital of Zhengzhou University, Zhengzhou, 450000 China

**Keywords:** Hepatocellular carcinoma, Cancer-associated fibroblasts, Stanniocalcin-1, Cancer stemness

## Abstract

**Background:**

Cancer-associated fibroblasts (CAFs), an important component of the tumor microenvironment (TME), play crucial roles in tumor stemness. It has been shown in various cancer studies that stanniocalcin-1 (STC1) is secreted by CAFs, however, its function in HCC is still not clear.

**Methods:**

The serum concentration and intracellular expression level of STC1 were quantified by ELISA and western blotting, respectively. The role of CAF-derived STC1 in HCC stemness was investigated by sphere formation, sorafenib resistance, colony formation, and transwell migration and invasion assays in vitro and in an orthotopic liver xenograft model in vivo. An HCC tissue microarray containing 72 samples was used to evaluate the expression of STC1 and Notch1 in HCC tissues. Coimmunoprecipitation (CoIP) and dual-luciferase reporter assays were performed to further explore the underlying mechanisms. ELISAs were used to measure the serum concentration of STC1 in HCC patients.

**Results:**

We demonstrated that CAFs were the main source of STC1 in HCC and that CAF-derived STC1 promoted HCC stemness through activation of the Notch signaling pathway. In HCC patients, the expression of STC1 was positively correlated with Notch1 expression and poor prognosis. The co-IP assay showed that STC1 directly bound to Notch1 receptors to activate the Notch signaling pathway, thereby promoting the stemness of HCC cells. Our data further demonstrated that STC1 was a direct transcriptional target of CSL in HCC cells. Furthermore, ELISA revealed that the serum STC1 concentration was higher in patients with advanced liver cancer than in patients with early liver cancer.

**Conclusions:**

CAF-derived STC1 promoted HCC stemness via the Notch1 signaling pathway. STC1 might serve as a potential biomarker for the prognostic assessment of HCC, and the stromal-tumor amplifying STC1-Notch1 feedforward signal could constitute an effective therapeutic target for HCC patients.

**Supplementary Information:**

The online version contains supplementary material available at 10.1186/s12967-023-04085-8.

## Background

As the fourth leading cause of cancer death, liver cancer imposes a heavy burden on global health [1]. Hepatocellular carcinoma (HCC), which accounts for approximately 80% of all primary liver cancers, is still difficult to treat [2]. Due to the high recurrence and metastasis rates of HCC, multidrug resistance and limitations of the current treatment plans, the survival rate of HCC patients is still low. An increasing number of studies have suggested that liver cancer stem cells (LCSCs) drive the progression, recurrence and metastasis of HCC through their self-renewal, differentiation, heterogeneity and drug resistance, and these cells are considered an important target subgroup for the successful treatment of HCC [3]. In-depth exploration of the mechanism that affects the stemness of HCC cells and the suppression and elimination of LCSCs is urgently needed for the successful treatment of HCC patients.

Studies have shown that the regulation and maintenance of tumor stemness depends on the tumor microenvironment (TME) and has clear plasticity [4], suggesting that there is crosstalk between the stroma and the tumor. The TME is a diversified, complex and integrated ecosystem composed of relatively differentiated tumor cells, cancer stem cells (CSCs), extracellular matrix, stromal cells and various cytokines and provides a supportive internal environment for tumor occurrence and development [5]. Adaptive changes in the TME are mainly driven by the activation of cancer-associated fibroblasts (CAFs) [6]. As the predominant stromal cells in the TME, CAFs can reprogram the TME and promote cancer progression by inducing angiogenesis, changing the matrix stiffness, regulating the immune cell response, and secreting exosomes, cytokines and secreted proteins [7, 8]. CAFs are associated with the poor prognosis of many kinds of tumors; thus, they have become potential targets for tumor treatment. Recent studies have reported that CAFs can maintain or promote cancer stemness in a variety of malignant tumors and reprogram cancer cells into cancer stem cells [9–12]. Studies have also shown that the crosstalk between CAFs and HCC cells promotes HCC stemness and progression, but the specific mechanism has not been fully defined [13]. Approximately 80% of HCC cases are accompanied by liver fibrosis or cirrhosis during their origin and development [14], which indicates that the accumulation of CAFs and fibrosis in the TME play an important role in the development and growth of HCC. Identifying the crosstalk between CAFs and HCC cells and delineating the specific molecular mechanism will help to optimize the effect of HCC targeted therapy.

Staniocalcin-1 (STC1), a glycoprotein, is associated with the proliferation of stem cells and progenitor cells [15] and can affect and regulate cancer stemness [16, 17]. In addition, abnormally high expression of STC1 has also been found in a variety of cancers and has been shown to promote the initiation and development of tumors [18, 19]. One recent study showed that STC1 could inhibit the phagocytosis of antigen-presenting cells (APCs), leading to tumor immune escape and immunotherapy resistance [20]. STC1, as a secreted protein, plays a role in the TME and is related to the stemness and malignant progression of various tumors. In studies of colorectal cancer, lung adenocarcinoma, and breast cancer, CAFs were shown to secrete STC1 to promote tumor progression [21–23]. However, in HCC, the relationship between STC1 and CAFs or the role of STC1 in HCC stemness has not been investigated.

The Notch signaling pathway is considered to be an ancient and highly conserved signaling pathway that regulates many cell processes in an environment-dependent manner [24]. After the Notch signaling pathway is activated, the Notch intracellular domain (NICD) is released and translocated to the nucleus to interact with the DNA-binding protein CSL (RBP-J) to regulate the transcription of its target genes [25]. CSL is a ubiquitous transcription factor (TF) that regulates gene expression after recruiting other co-TFs, and its Su(H) motif largely determines the target gene of the Notch signaling pathway [26]. We and others have shown that the Notch signaling pathway is involved in cancer stemness maintenance and regulation [27–30]. Recent studies have found that the Notch signaling pathway also plays an important role in the regulation of cancer stemness by the TME, including in HCC [31], but its mechanism needs further exploration. Notch overactivation was found in ~ 30% of HCCs and represents a molecular subtype of HCC with unique histology and prognosis [32]. At present, some drugs targeting the Notch signaling pathway have been tested in phase I/II clinical trials, but the therapeutic effects were limited. Thus, it is very important to explore how the Notch signaling pathway is overactivated and regulates stemness in tumors.

In this study, we confirmed that CAF-secreted STC1 promoted HCC stemness and provided evidence for an amplifying STC1-Notch1 axis in HCC stromal-tumor crosstalk. These findings suggested that the STC1-Notch1 axis might be a new therapeutic target for HCC. Furthermore, serum STC1 might be a promising biomarker for the prognostic evaluation of HCC.

## Methods

### Bioinformatics analysis

We downloaded the transcriptomic data and the clinicopathological data of the TCGA-LIHC project from The Cancer Genome Atlas database at http://portal.gdc.cancer.gov/. Relevant survival data were gotten from previous literature [33]. Gene Set Enrichment Analysis (GSEA) and Gene Ontology (GO) enrichment analysis were performed on these data using “clusterProfiler” R packages [34]. For survival analysis, we excluded samples with an overall survival time of fewer than 30 days to exclude non-tumor causes of death. Kaplan–Meier survival analysis was performed with the log-rank test.

### Clinical liver samples

Samples of HCC tissue and the corresponding adjacent normal HCC tissues were collected from HCC patients who had undergone surgical resection and had not received radiotherapy and chemotherapy. We also collected 5 samples of hepatic hemangioma. The collection and usage of human specimens were approved by the Ethics Committee of Tongji Hospital, HUST, Wuhan, China (IRB ID: TJ-IRB20220562).

### Immunohistochemistry (IHC)

IHC staining with the STC1 antibody (HPA 023918) and Notch1 (Abcam, ab52627) was performed to detect the protein expression level in HCC tissues. The IHC staining score was carried by the ImageJ IHC profiler (http://sourceforge.net/projects/ihcprofiler). The staining intensity and staining range were used to evaluate the IHC score. The staining intensity was divided into 0 points (negative), 1 point (weakly positive), 2 points (moderately positive), and 3 points (strongly positive). The staining range (n%) was determined according to the percentage of positive areas in total areas. The product of staining intensity and staining range is the protein expression score. The score ≤ 30 was determined as low expression, while the score > 30 was determined as high expression, and then the subsequent survival analysis was performed. Three pathologists were invited to reassess the IHC staining results independently without informing the patients of any clinical information.

### Isolation and purification of CAFs and normal fibroblasts (NFs)

The primary CAFs and NFs were isolated from the clinical HCC tissues and liver tissues adjacent to hepatic hemangioma respectively. The fresh liver tissues were washed with D-Hank’s solution, cut into 2–3 mm fragments, and then plated in a culture dish with DMEM (GIBCO) comprising 15% fetal bovine serum (FBS) for attachment. Fibroblasts were allowed to grow out of tumor fragments for 1–2 weeks. 95% purified fibroblasts were obtained after 2–3 generations and verified by the fibroblast marker α-SMA.

### Cell culture and reagents

MHCC-97H was purchased from the Cell Bank of the Chinese Academy of Sciences (Shanghai, China) and cultured in DMEM comprising 10% FBS. SNU-398 was obtained from the American Type Culture Collection (ATCC, Manassas, VA, USA), and cultivated in RPMI 1640 Medium (GIBCO) with 10% FBS. The recombinant human STC1 protein (rhSTC1) and STC1 neutralizing antibody (STC1-Ab) were purchased from R&D Systems. And RO4929097 was purchased from MedChemExpress (MCE).

### Immunofluorescence (IF)

Cells were fixed with 4% paraformaldehyde, washed with PBS 3 times, permeabilized with 0.1% Triton X-100 for 10 min, and incubated in 10% normal fetal sheep serum in PBS for 40 min at room temperature. Then the CAFs and NFs were co-incubated with α-SMA (Boster, BM0002) and STC1 (Santa Cruz, sc-293435) primary antibodies at 4 °C overnight. The MHCC-97H were incubated with Notch1 (Abcam, ab52627) and/or STC1 primary antibodies. Observed and photographed cells under the fluorescence microscope.

### ELISA

The culture medium supernatants from CAFs, NFs, and HCC cell lines (MHCC-97H and SNU-398) were collected and centrifuged at 1500 rpm for 5 min. For plasma samples, cells were removed by centrifugation at 3000 rpm for 10 min. Subsequently, the secretion of STC1 was detected according to the manufacturer’s instructions using the ELISA kit (Boster, EK1404).

### Western blot and qRT-PCR

The cells were given an appropriate amount of RIPA /PMSF/cocktail buffer and centrifuged to extract the protein. Then the total proteins were separated on SDS-PAGE and transferred onto the PVDF membrane (Merck Millipore, USA). After blocking with 5% milk for 1 h, the PVDF membranes were incubated with primary antibodies overnight at 4 °C. Anti-β- actin (Proteintech, 66009-1-ig) was used as a loading control. The primary antibodies were as follows: STC1 (Santa Cruz), NANOG (Cell Signaling Technology, 4903), OCT4 (CST, 2750), SOX2 (CST, 3579), Notch1 (CST, 3608), cleaved Notch1 (CST, 4147), HES1 (CST, 11988), and HEY1 (ABclonal, A16110). The ImageJ software (NIH, America) was used to quantify the gray value of the protein bands.

Trizol reagent (Invitrogen, Carlsbad, CA, USA) and HiScript® III Reverse Transcriptase (Vazyme, Jiangsu, China, R302-01) were used to extract the ribonucleic acid (RNA) and reversed to cDNA following the manufacturer’s instructions. Then quantitative real-time PCR was carried out using ChamQ Universal SYBR qPCR Master Mix (Vazyme, Q711-02). The data were analyzed by the 2-ΔΔCt (Ct, cycle threshold) method. Primer sequences were provided in Additional file 4: Table S1.

### Sphere formation assay

The cells were seeded in 24-well low attachment plates (Corning, NY, USA) with tumor sphere medium, composed of DMEM/F12 medium with 1% penicillin/streptomycin, 1 × B27 supplement (cat# 17504-044; GIBCO), 20 ng/ml EGF (cat# PHG0311; GIBCO), 10 ng/ml bFGF (cat# PHG0266; GIBCO), and 1% methyl cellulose (cat# M0262; Sigma-Aldrich). Fresh medium was added every 3–4 days. Spheres were visualized and counted by microscopy.

### CCK8 toxic assay

The Cell Counting Kit (CCK-8) (40203ES92, Yeasen, Shanghai, China) was used to measure the sensitivity of the cells to Sorafenib. 1000 cells per well were seeded in 96-well plates, and after cell adhesion, treated with different concentrations (2.5, 5, 10, or 20 μM) of Sorafenib (Sigma-Aldrich). After incubating for 24 h at 37 ℃, CCK-8 reagent was added to each well. 2 h later, an absorption value at 450 nm was detected at the microplate reader (Thermo Fisher, Shanghai, China).

### Colony-formation assay

Cells were seeded at a density of 1000 cells per well in 6-well plates. Approximately 10 days later, colonies were dyed with crystal violet (C0121, Beyotime, China) and counted after fixing the cells with 4% methanol for 20 min. The results are presented as the mean ± SD.

### Transwell migration and invasion assays

For the migration assay, the serum-free cell suspension was added into the upper transwell chambers (Millipore, Billerica, MA, USA) without FBS, and 600 µl of DMEM with 10% FBS was added as a chemoattractant. And cell invasion assay was performed with Matrigel (1:8 diluent, BD Biosciences, San Jose, CA, USA) coating the transwell chambers. After 24 h (migration) or 32 h (invasion) of incubation, the membranes were fixed and imaged. The number of cells in 3 randomly selected was counted, and the experiments were repeated three times.

### Flow cytometry analysis

Cells were washed by PBS once after digestion and centrifugation. For cell cycle, cells needed to be dissociated into single cells, fixed with 70% cold ethanol, and stained with PI (KeyGEN BioTECH, KGA512). For cell apoptosis, cells were incubated according to the steps of the cell apoptosis kit (Lianke Biotech, AP101). The stained cells were analyzed with the BD machine and FlowJo software.

### Virus infection

Viral particles expressed STC1 shRNA, STC1, or Notch1 shRNA were obtained from DesignGene Biotechnology (Shanghai, China) and Genechem Corporation (Shanghai, China). The sequences for virus-based RNAi were provided in Additional file 4: Table S1. CAFs or HCC cells were infected with lentivirus. 48 h after infection, puromycin was added to screen cells for establishing stable cell lines.

### Co-immunoprecipitation (Co-IP)

Cells were collected and lysed in an appropriate precooled IP buffer. The cell lysate was centrifuged after being frozen for 30 min. Took 50 µl lysate supernatant as input. And the rest was incubated with the indicated antibody on a rotating device overnight at 4 ℃. The next day, protein A/G Magnetic Beads (MCE, HY-K0202) were washed 3 times with PBST and added to the cell supernatant-antibody system. After incubation on a rotating device at 4 ℃ for 4 h, the magnetic beads were washed 6 times and boiled. Samples were conducted by Western blot analysis. The primary antibodies had been listed in Western blot.

### Dual-luciferase reporter assay

The STC1 promoter fragment was inserted into the pGL3-Basic vector. The stable lines overexpressed NICD and the control cells were co-transfected with STC1 reporter-gene plasmid and pGMLR-TK. Following a 48 h incubation, a Dual-Luciferase Reporter Assay Kit (Yeasen, 11402ES60) was employed to measure firefly luciferase activity, and Renilla luciferase activity was implemented for normalization.

### Mouse orthotopic liver xenograft tumor model

Four-week-old male nude mice were bought from GemPharmatech company and raised in a pathogen-free barrier environment. HCC cells with or without CAFs were resuspended in PBS and slowly injected into the left lobe of the liver. Animals were sacrificed about 4–5 weeks after implantation. Bioluminescence was measured after an intraperitoneal injection of 100 µl of potassium d-luciferin salt (30 mg/ml, per animal). All experiments were approved by Tongji Hospital Institutional Review Board (IRB ID: TJH-202206023).

### Statistical analysis

Each assay was carefully performed with three independent experiments, and the data were shown as the mean and standard deviation (mean ± SD) unless otherwise indicated. Statistical analysis was processed using R (v4.0.2) and GraphPad Prism 9.0 software. We chose appropriate statistical tests according to the types of data. For the data approximately normally distributed, student t-test and one-way ANOVA were performed. Wilcoxon signed-rank test was applied to analyze the nonnormal distribution data. Survival analysis was evaluated using a log-rank test. P < 0.05 was regarded as statistically significant.

## Results

### CAF-derived STC1 promotes HCC stemness

**Fig. 1:**
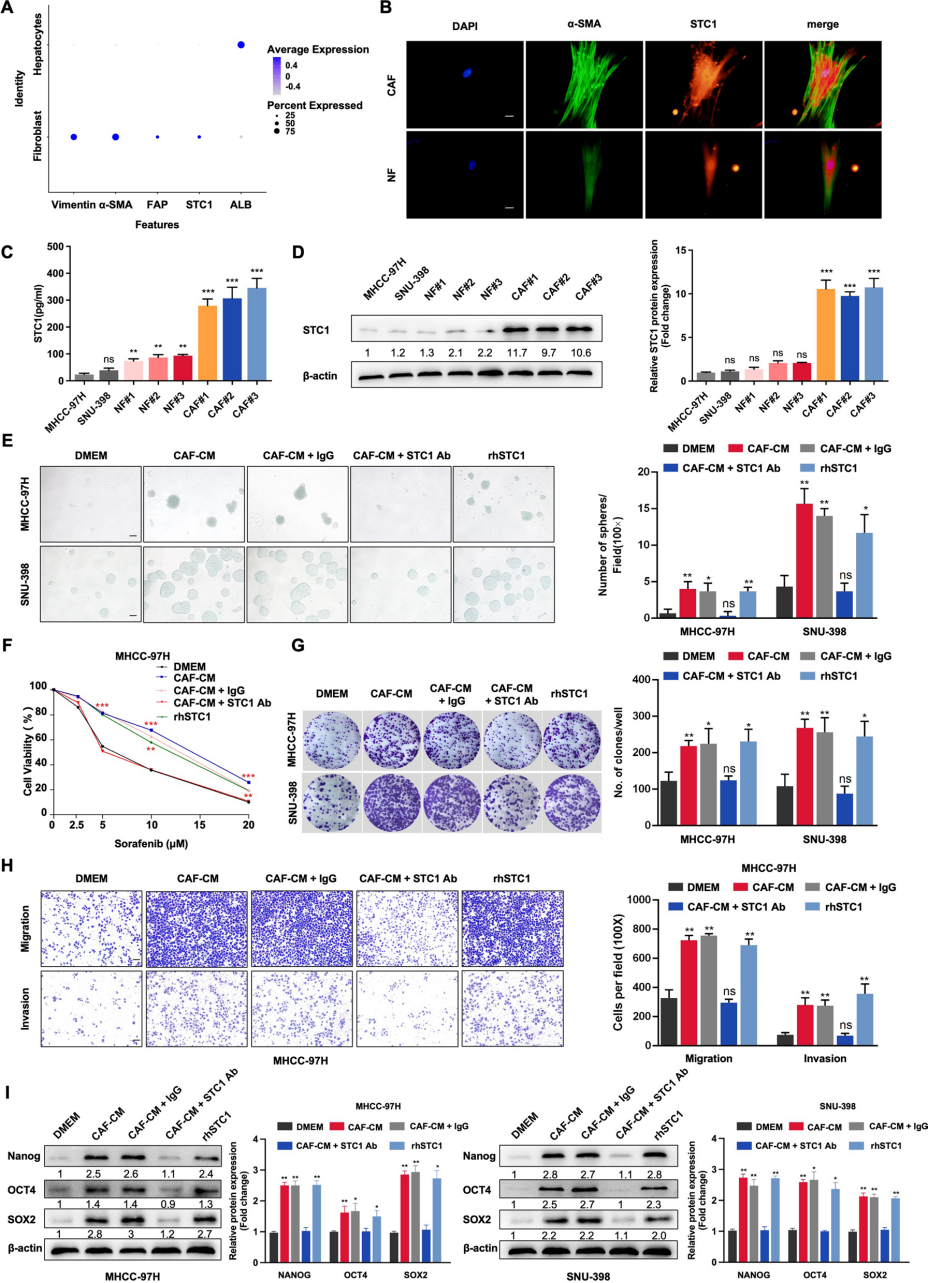
CAF-derived STC1 promotes HCC stemness. **A** Analysis of HCC single-cell sequencing data published to compare the expression levels of STC1 in HCC cells and fibroblasts. **B**. Representative images of IF staining for α-SMA and STC1 in CAFs and NFs. Scale bar, 20 μm. **C**, **D** The secreted concentrations and intracellular expression levels of STC1 in CAFs, NFs, MHCC-97H cells, and SNU-398 cells were measured by ELISA (**C**) and western blot analysis (**D**). **E** The sphere formation assay revealed the self-renewal ability of MHCC-97H and SNU-398 cells treated with different culture media. HCC cells were treated with DMEM, CAF-CM, CAF-CM + IgG, CAF-CM + STC1-Ab (1 μg/ml) or rhSTC1 (20 ng/ml). Scale bar, 100 μm. **F** MHCC-97H cells in different groups were treated with sorafenib for 24 h, and cell viability was assessed by a CCK8 toxicity assay. **G** The colony formation assay showed the proliferation abilities of the indicated cells. **H** Transwell migration and invasion assays showed the migration and invasion abilities in different MHCC-97H cell groups. Scale bar, 100 μm. **I** Measurement of the expression levels of cancer stemness markers, including NANOG, OCT4, and SOX2, in MHCC-97H and SNU-398 cells treated with different culture media. For the statistical analysis, ns, no significance, *P < 0.05, **P < 0.01, and ***P < 0.001, t test

CAFs were isolated from HCC patient tumor tissue samples, and NFs were purified from liver tissues adjacent to hepatic hemangiomas. To determine whether CAFs were the main source of STC1 in HCC, we first analyzed the published single-cell sequencing data of HCC [35] and found that STC1 was more highly expressed in fibroblasts than in HCC cells (Fig. 1A). IF showed higher abundances of α-smooth muscle actin (α-SMA) (green) and STC1 (red) in CAFs than in NFs. α-SMA and STC1 colocalized in the cytoplasm and nucleus (Fig. 1B). Next, we individually measured the secreted and intracellular levels of STC1 in the CAFs, NFs, MHCC-97H, and SNU-398. ELISA showed that CAFs secreted the greatest amount of STC1 (Fig. 1C). The protein expression of STC1 was dramatically upregulated in CAFs relative to that in other cells (Fig. 1D). Collectively, these data indicated that CAFs were the main source of STC1 in the HCC TME.

Our previous study demonstrated that CAFs could promote or maintain stem-like properties in HCC [31, 36]. To investigate whether CAF-derived STC1 affects HCC stemness, MHCC-97H and SNU-398 cells were cultured in different media: DMEM, conditioned medium of CAFs (CAF-CM), CAF-CM supplemented with 1 μg/ml IgG (CAF-CM + IgG), CAF-CM supplemented with the STC1 neutralizing antibody (1 μg/ml, CAF-CM + STC1-Ab), and DMEM supplemented with recombinant human STC1 protein (rhSTC1, 20 ng/ml). Then, a series of assays were performed to test the stem-like properties of HCC cells. The sphere formation assay showed that CAF-CM or rhSTC1 significantly enhanced the self-renewal ability of MHCC-97H and SNU-398 cells, while the STC1-Ab dramatically reversed the increase in the self-renewal ability induced by CAF-CM (Fig. 1E). Different concentrations of sorafenib were used to treat HCC cells for 24 h. The data indicated that the CAF-CM, CAF-CM + IgG, and rhSTC1 groups exhibited higher sorafenib resistance than the DMEM and CAF-CM + STC1-Ab groups (Fig. 1F and Additional file 1: Fig. S1A). The colony formation assay demonstrated that either CAF-CM or rhSTC1 could promote proliferation, while the STC1 neutralizing antibody attenuated the promoting effects of CAF-CM (Fig. 1G). Moreover, transwell migration and invasion assays showed that after treatment with CAF-CM or rhSTC1, MHCC-97H and SNU-398 cells had higher migration and invasion abilities than cells treated with DMEM. Compared with the CAF-CM group, the CAF-CM + STC1-Ab group had diminished migration and invasion abilities (Fig. 1H and Additional file 1: Fig. S1B). CAFs and STC1 could upregulate the expression of cancer stemness genes (NANOG, SOX2, and OCT4), and STC1-Ab treatment blocked the promoting effect of CAFs on the expression of stemness genes in HCC cells (Fig. 1I). To explore the relationship between CAF-derived STC1 and cell cycle, we performed GSEA on the HCC samples in the TCGA database. The results revealed that the co-high expression of CAFs and STC1 was positively correlated with positive regulation of cell cycle and G1/S cell cycle (Additional file 1: Fig. S1C). And the high expression of STC1 in HCC was related to positive regulation of the G1/S cell cycle signaling pathway (Additional file 1: Fig. S1D). Flow cytometry was performed to analyze the cell cycle and apoptosis. We found that CAFs and STC1 decreased the proportion of cells in the G1 phase, and increased the proportion of cells in the S phase, which suggested that CAFs and STC1 could promote the G1-S transition of HCC cells (Additional file 1: Fig. S1E). After co-culture of CAF or stimulation of rhSTC1, there was no significant difference in the apoptosis of SNU-398 cells (Additional file 1: Fig. S1F). However, the apoptosis of SNU-398 cells was increased after treatment with sorafenib (5 μM), while CAF-CM and rhSTC1 inhibited the apoptosis (Additional file 1: Fig. S1G). Together, these data suggested that CAFs were the main source of STC1 in the HCC TME, and that CAFs and CAF-derived STC1 could promote self-renewal, sorafenib resistance, proliferation, migration, invasion, and G1-S transition of HCC cells and protect HCC cells from sorafenib-induced apoptosis.

### Knocking out STC1 in CAFs attenuated their ability to promote HCC stemness

**Fig. 2:**
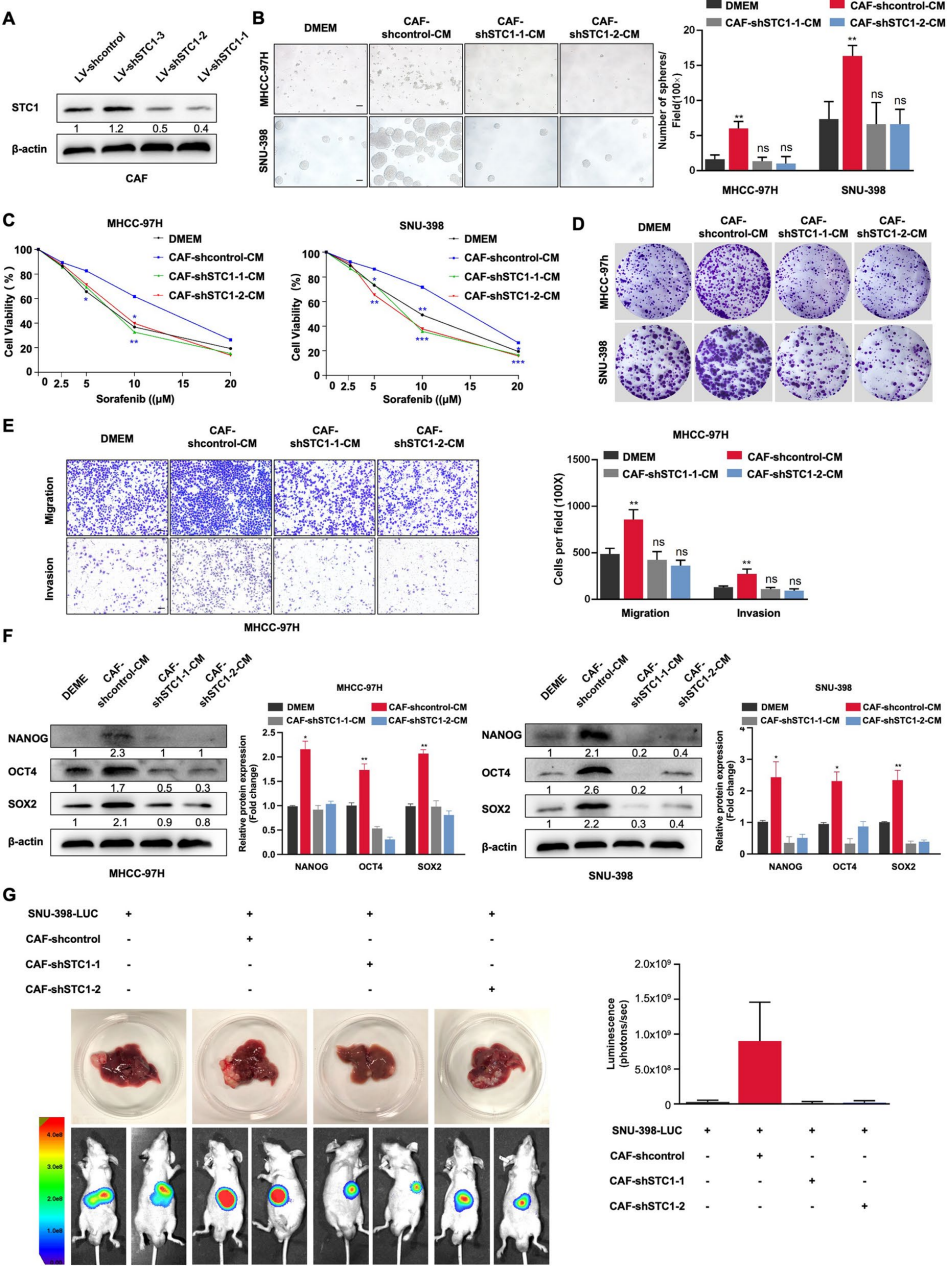
Knocking out STC1 in CAFs attenuated their ability to promote HCC stemness.** A** The selection of shRNA sequences and the regulation of STC1 in CAFs. **B** A sphere formation assay was used to analyze the self-renewal ability of HCC cells. MHCC-97H and SNU-398 cells were treated with DMEM, CAF-shcontrol-CM, CAF-shSTC1-1-CM, or CAF-shSTC1-2-CM. Scale bar, 100 μm. **C** HCC cells were treated with different conditioned medium of CAFs for 48 h, and their viability was assessed after 24 h of sorafenib treatment. **D** A colony formation assay was performed in the indicated cells. **E** Representative images of transwell migration and invasion assays of different groups of MHCC-97H cells. Scale bar, 100 μm. **F** Western blot analysis of the expression of cancer stemness markers in the indicated HCC cells. **G** HCC cells with or without the indicated CAFs were transplanted into the livers of nude mice. Representative bioluminescence images of each group are shown. For the statistical analysis, ns, no significance, *P < 0.05, **P < 0.01, and ***P < 0.001, t test

Primary CAFs were used to establish the stable cell lines CAF-shcontrol, CAF-shSTC1-1, and CAF-shSTC1-2 via lentiviral transduction (Fig. 2A and Additional file 1: Fig. S1H). The data showed that downregulation of STC1 in CAFs significantly decreased the CAF-enhanced self-renewal ability and the CAF-enhanced resistance to sorafenib ability of HCC cells (Fig. 2B and C). In addition, cells treated with CAF-shSTC1-1-CM and CAF-shSTC1-2-CM possessed lower proliferation, migration, and invasion abilities than those treated with CAF-shcontrol-CM (Fig. 2D and E, Additional file 1: Fig. S1I and J). Western blot analysis revealed that downregulation of STC1 in CAFs significantly reversed the CAF-mediated upregulation of NANOG, SOX2, and OCT4 expression in HCC cells (Fig. 2F). Additionally, we explored the effect of CAF-derived STC1 in vivo in the mouse orthotopic liver xenograft model. SNU-398 luciferase-tagged (SNU-398-LUC) HCC cells mixed with or without CAFs subjected to different treatments were injected into the livers of nude mice. The mice were randomly divided into four groups: SNU-398-LUC cell group, SNU-398-LUC cell + CAF-sh control group, SNU-398-LUC cell + CAF-shSTC1-1 group, and SNU-398-LUC cell + CAF-shSTC1-2 group. Twenty-eight days after cell injection, the tumor size was measured by bioluminescence imaging. Interestingly, we observed that the luminescence intensity in the SNU-398-LUC cell + CAF-sh control group was higher than that in the SNU-398-LUC cell group, while knockdown of STC1 in CAFs attenuated the luminescence intensity (Fig. 2G). Taken together, these data indicated that STC1 played a vital role in CAF-mediated promotion of HCC stemness in vitro and in vivo.

### CAF-derived STC1 promoted HCC stemness in a Notch1-dependent manner

**Fig. 3:**
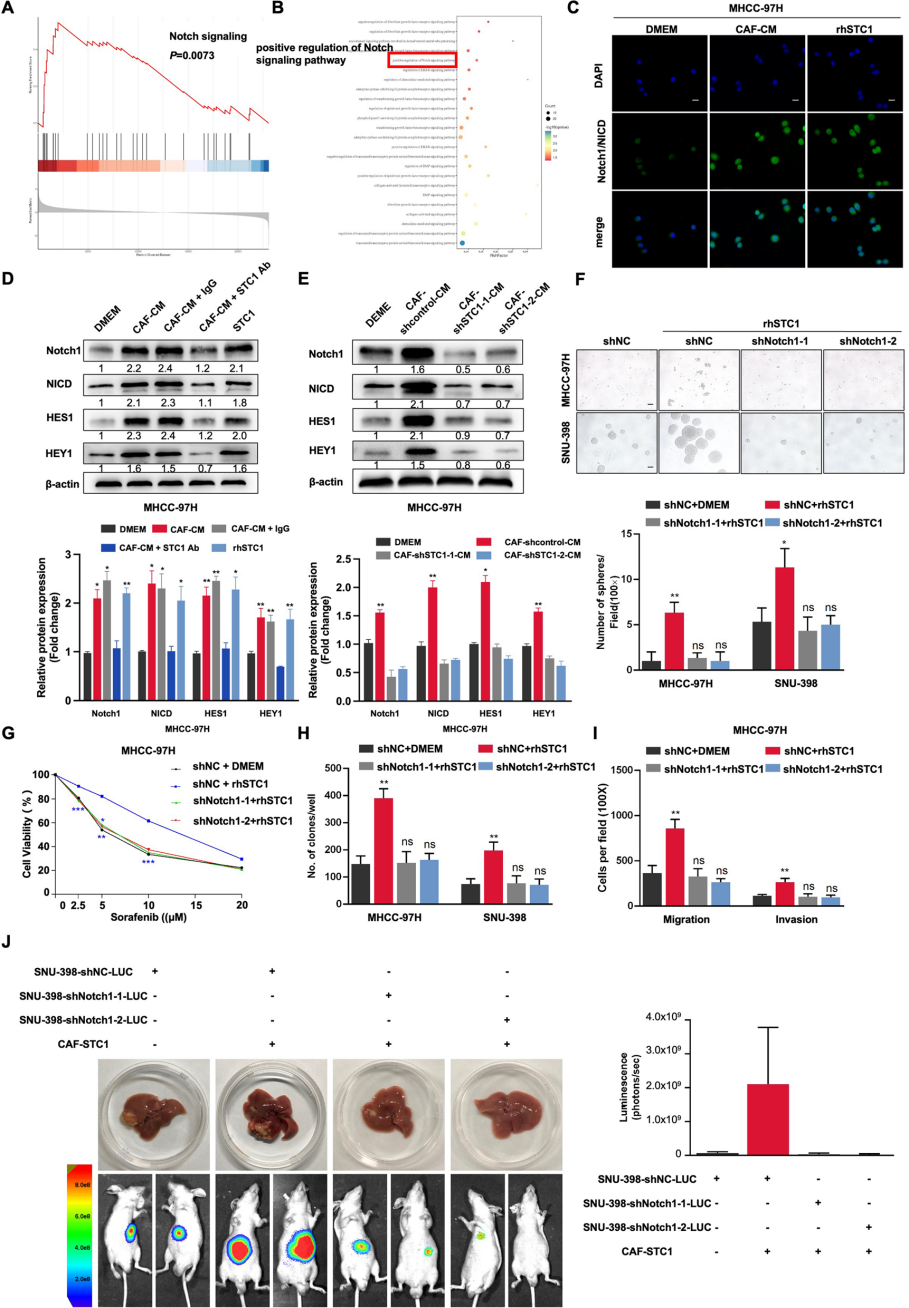
CAF-derived STC1 promoted HCC stemness in a Notch1-dependent manner. **A**, **B** GSEA (**A**) and GO analysis (**B**) of the HCC samples in the TCGA database were performed. **C** Immunofluorescence images of MHCC-97H cells cultured with DMEM or rhSTC1 (20 ng/ml) to assess the localization of Notch1/NICD (green), Scale bars, 50 μm. **D**, **E** The protein levels of Notch1, NICD, HES1, HEY1, and STC1 were measured by western blotting. β-Actin was used as the loading control. **F** A sphere formation assay was used to determine the self-renewal ability of MHCC-97H and SNU-398 cells with Notch1 knockdown in the presence of DMEM or rhSTC1 (20 ng/ml). Scale bar, 50 μm. **G** After treatment with sorafenib for 24 h, the cell viability of different groups was assessed by a CCK8 toxicity assay. **H** The proliferation ability of different groups of HCC cells was evaluated by colony formation assay. **I** A Transwell migration assay was performed to detect the migration ability of the indicated MHCC-97H cells. Scale bar, 100 μm. **J** SNU-398-LUC cells with knockdown of Notch1 were mixed with stable CAF-STC1 cells lines and implanted in the livers of nude mice. The bioluminescence intensity of the groups was evaluated. For the statistical analysis, ns, no significance, *P < 0.05, **P < 0.01, and ***P < 0.001, t test

The Notch signaling pathway is closely related to cancer stemness. Recent studies have shown that the Notch signaling pathway plays an important role in the regulation of tumor stemness by the TME. Our previous studies demonstrated that the Notch1 signaling pathway is important for promoting stem-like properties in HCC [27–29]. To explore the underlying mechanism by which CAF-derived STC1 promoted HCC stemness, we performed GSEA and GO analysis on the HCC samples in the TCGA database. The results indicated that high expression of STC1 was significantly related to positive regulation of the Notch signaling pathway (Fig. 3A and B). IF showed higher Notch1/NICD (green) expression in CAF-CM- and rhSTC1-treated MHCC-97H cells than in DMEM-treated cells (Fig. 3C). Next, we evaluated the expression of downstream molecules of the Notch1 signaling pathway, and we found that in the CAF-CM, CAF-CM + IgG, and rhSTC1 groups, the NICD, HES1, and HEY1 expression levels were significantly higher than those in the DMEM and CAF-CM + STC1 Ab groups (Fig. 3D and Additional file 2: Fig. S2A). When STC1 was knocked out in CAFs, the promoting effect of CAFs on the expression of Notch1 signaling pathway molecules in HCC cells was inhibited (Fig. 3E and Additional file 2: Fig. S2B). These data suggested that the Notch signaling pathway is downstream of STC1.

To further explore whether Notch1 was involved in CAF-derived STC1-mediated HCC stemness, the expression of Notch1 was knocked down at the mRNA level (via lentiviral transduction) (Additional file 2: Fig. S2C and D) and inhibited at the protein level (via RO4929097 treatment) (Additional file 3: Fig. S3A) in HCC cells. Our previous research proved that the CAF-induced promotion of HCC stemness was weakened after Notch1 was inhibited [31]; thus, in this part, we focused our attention mainly on the effect of Notch1 on CAF-derived STC1-induced stem cell-like properties. The data revealed that the self-renewal ability of shNotch1 cells was attenuated regardless of rhSTC1 treatment (Fig. 3F). Similarly, after Notch1 knockdown, despite treatment with rhSTC1, the resistance of HCC cells to sorafenib was diminished (Fig. 3G and Additional file 2: Fig. S2E). Additionally, knockdown of Notch1 in HCC cells decreased their proliferation, migration, and invasion abilities despite pretreatment with rhSTC1 (Fig. 3H, I, and Additional file 2: Fig. S2F–H). The in vivo orthotopic liver xenograft model showed that knockdown of Notch1 decreased the luminescence intensity (Fig. 3J). Consistent with this finding, treatment with RO4929097 (RO), a γ-secretase inhibitor, inhibited the ability of STC1 to promote the stemness properties of HCC cells and decreased the expression of stemness genes in HCC cells (Additional file 3: Fig. S3B–F). Taken together, these data suggested that the effect of CAF-derived STC1 on HCC stemness was prominently suppressed through Notch1 knockdown or inhibition, indicating that the Notch1 signaling pathway plays an important role in the process by which CAF-secreted STC1 promotes HCC stemness.

### CAF-secreted STC1 directly bound to Notch1 receptors and activated the Notch1 signaling pathway

**Fig. 4:**
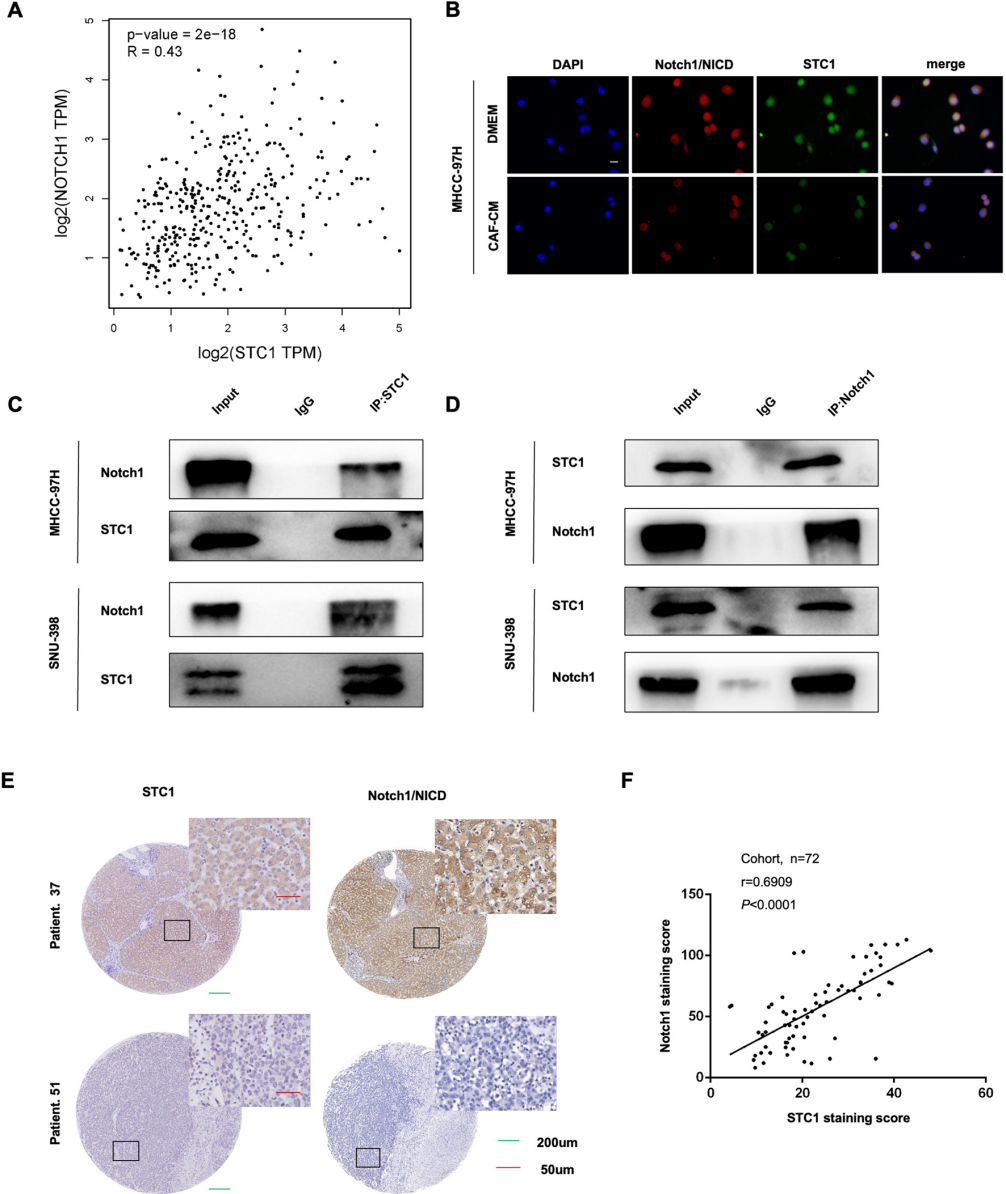
CAF-secreted STC1 directly bound to Notch1 receptor and activated the Notch signaling pathway. **A** Correlations between STC1 and Notch1 expression in HCC TCGA database were determined by as Pearson correlation analysis; **B** MHCC-97H cells were cultured with CAF-CM or DMEM. Immunofluorescence images of cells showing the localization of STC1 (red) and Notch1 (green) after 48 h of pretreatment. Scale bar, 50 μm; **C**, **D** Reciprocal co-IP experiments were performed with an anti-STC1 or anti-Notch1 antibody in MHCC-97H (**C**) and SNU-398 (**D**) cells. The interaction of endogenous STC1 and endogenous Notch1 was verified by immunoblotting. **E** The expression of STC1 and Notch1 in tumor tissues and adjacent nontumor tissues on tissue microarrays was analyzed by IHC staining. **F** Correlation between STC1 and Notch expression in HCC tissues. Scale bars: 200 μm and 50 μm. **** *P* < 0.0001, Pearson correlation analysis

To investigate the underlying mechanism of STC1-mediated activation of Notch1 signaling, we first analyzed the correlation between STC1 and Notch1 expression in the HCC TCGA database at the GEPIA website (http://gepia.cancer- pku.cn/). The result prompted that STC1 was positively correlated with Notch1 (Fig. 4A). IF showed the colocalization of STC1 (green) and Notch1 (red) in MHCC-97H cells (Fig. 4B). Then, co-IP experiments were performed in MHCC-97H and SNU-398 cells. The results proved that there is a physical interaction between STC1 and Notch1 (Fig. 4C and 4D) and were consistent with the results of a previous study, which confirmed that STC1 could directly bind to Notch1 as a noncanonical Notch ligand to activate the Notch1 signaling pathway [37]. To evaluate the possible correlation of STC1 and Notch1 expression in human HCC tissues, the protein levels of STC1 and Notch1 were evaluated by IHC staining on a tissue microarray (Fig. 4E). Importantly, we observed that STC1 expression was positively correlated with Notch1 expression in HCC patients (Fig. 4F; n = 72, r = 0.6909, **** *P* < 0.0001, Pearson correlation analysis). These data implied that CAF-secreted STC1 could activate the Notch1 signaling pathway by directly binding to Notch1, and in the clinical HCC samples we collected, STC1 expression was positively correlated with Notch1 expression.

### Notch1 directly regulated STC1 expression to establish an amplifying STC1-Notch1 feedforward signal

**Fig. 5:**
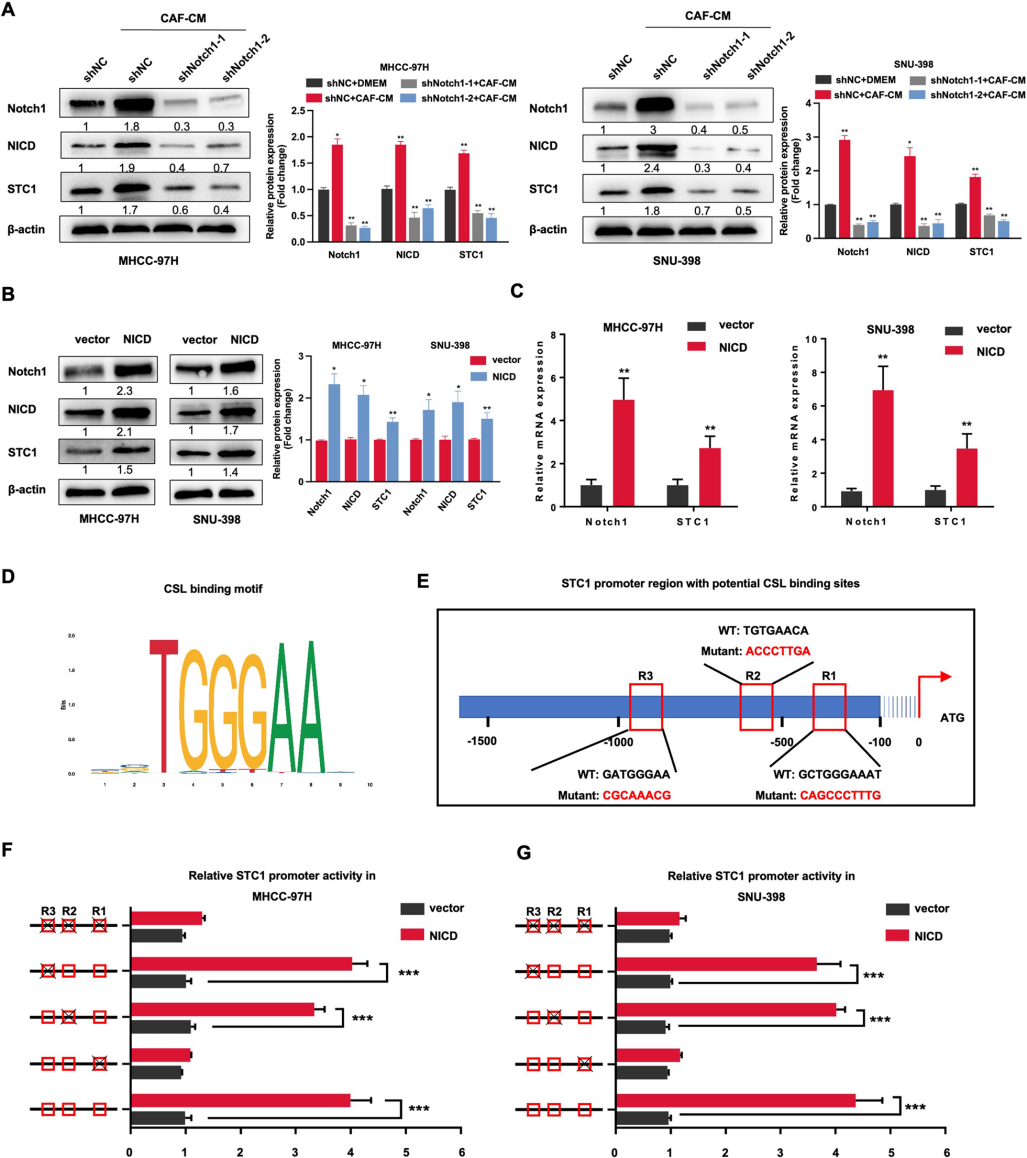
Notch1 directly regulated STC1 expression to establish a paracrine amplifying STC1-Notch1 feedforward signal. **A** Notch1 was knocked out in HCC cells, and western blotting was used to measure the expression levels of Notch1, NICD, and STC1. **B**, **C** Western blot (**B**) and qRT‒PCR (**C**) analyses were used to quantify the expression of Notch1 and STC1 in MHCC-97H and SNU-398 cells. **D** The CSL binding motif. **E** Mutation of possible binding sites of CSL and STC1 promoter sequences. **F**, **G** Luciferase reporter assays were performed to measure luciferase activities in MHCC-97H (**F**) and SNU-398 (**G**) cells. Renilla luciferase activity was used as the internal reference. For the statistical analysis, *P < 0.05, **P < 0.01, and ***P < 0.001, t test

In the previous experiment, we surprisingly found that CAF-CM-treated HCC cells had higher STC1 expression than DMEM-treated HCC cells by the IF experiment (Fig. 4B). In addition, we observed that Notch1 depletion reduced the protein expression of STC1 in HCC cells (Fig. 5A). This finding prompted us to examine whether STC1 expression was potentially regulated by the NICD at the transcriptional level in HCC cells. The STC1 protein and mRNA levels were increased by overexpressing NICD via lentiviral transduction in MHCC-97H and SNU-398 cells (Fig. 5B and C). NICD, the activated component of the Notch receptor, is translocated into the nucleus as a transcriptional coactivator and interacts with the DNA-binding protein CSL (RBP-J), a molecule that largely determines the target gene of the Notch signaling pathway. We obtained the CSL binding motif from the JASPAR database (http://jaspardev.genereg.net/) (Fig. 5D). Then, the STC1 promoter region was screened for possible CSL binding sites with JASPAR, and we chose the 3 most likely CSL binding sites for mutation (Fig. 5E). The dual-luciferase reporter assay suggested that CSL directly bound to the STC1 promotor and the mutation of R1 (binding region 1, -152/-143 bp) in the STC1 promoter abolished the promoter inducibility mediated by NICD overexpression (Fig. 5F and G). Collectively, these findings indicated that STC1 was regulated by Notch1 at the transcriptional level, generating an amplifying STC1-Notch1 feedforward signal in tumor-stromal crosstalk.

### STC1 was correlated with poor prognosis of HCC patients and might serve as a biomarker

**Fig. 6:**
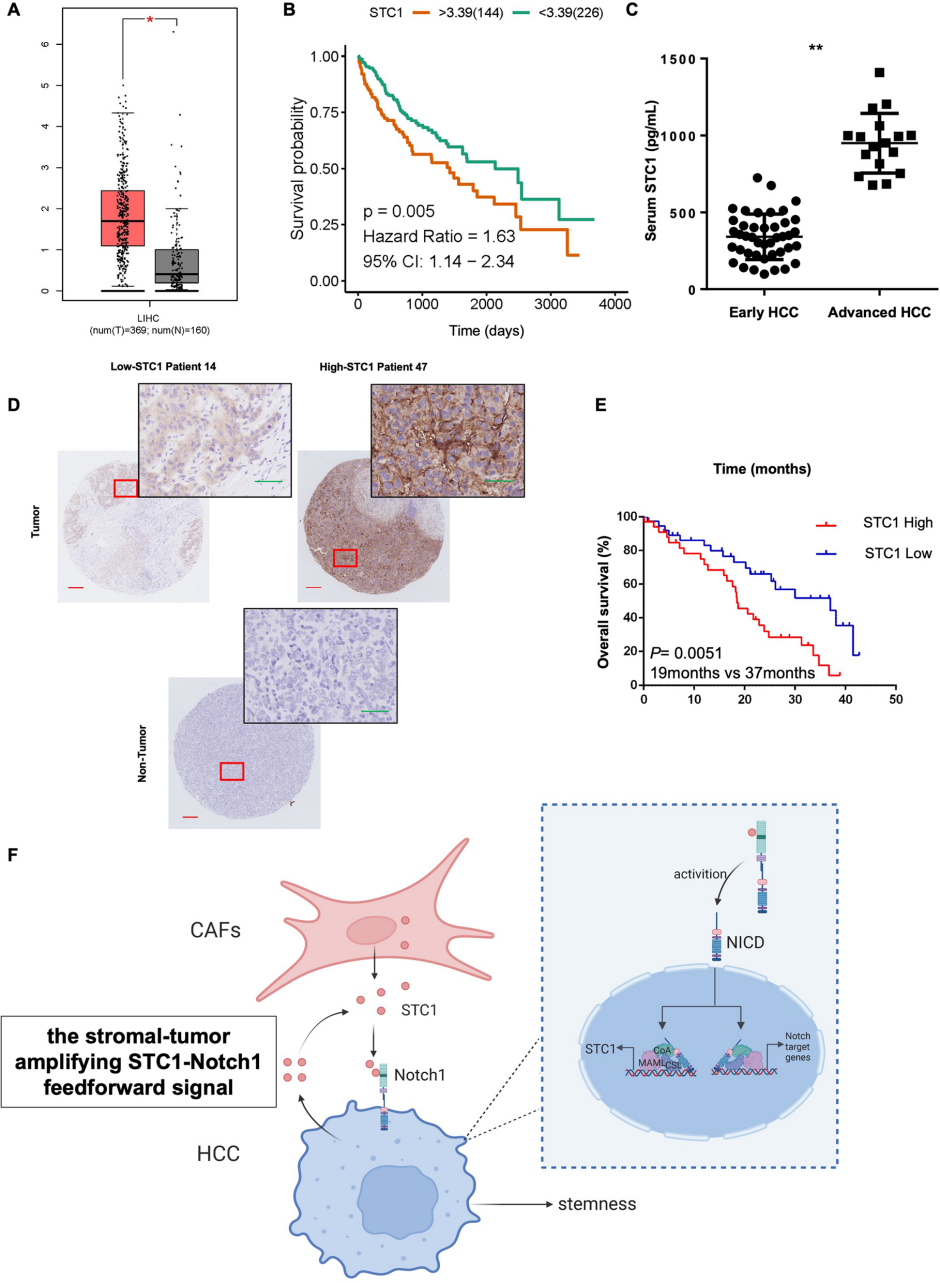
**S**TC1 was correlated with poor prognosis in HCC patients and might serve as a biomarker. **A** GEPIA analysis revealed that STC1 expression was upregulated in HCC. **B** Kaplan‒Meier analysis of overall survival was performed on the TCGA LIHC dataset with stratification according to the STC1 expression level. **C** Serum STC1 concentrations in patients with early-stage tumors (n = 42) and advanced-stage tumors (n = 17), as measured by ELISA (t test, **P < 0.01). **D** Representative images of IHC staining of STC1 in tumor and nontumor tissues. Scale bars: 200 μm and 50 μm. **E** The overall survival rate was compared according to the expression level of STC1 in HCC tissues. Patients with high STC1 expression had shorter overall survival times (19 months vs. 37 months, log-rank test, n = 72, ** P = 0.0051). **F** Schematic diagram of our study

The effect of CAF-derived STC1 on HCC cell stemness properties motivated us to explore the importance of STC1 expression in HCC patients. By using publicly available TCGA databases (GEPIA), we found that HCC tumor tissues had higher expression levels of STC1 than normal liver samples (Fig. 6A). In addition, high expression of STC1 predicted poor survival in HCC patients, as determined by Kaplan‒Meier analysis (Fig. 6B). During our analysis, samples from patients with an overall survival time of less than 30 days were excluded to exclude the effects of non-tumor-related causes of death. As STC1 is a secretory protein, we further investigated whether the concentration of STC1 in serum could serve as a clinical biomarker for HCC. We measured the concentration of STC1 in the serum of patients with advanced liver cancer (n = 17) and patients with early liver cancer (n = 42) by ELISA. Patients with advanced liver cancer possessed higher serum STC1 concentrations than patients with early liver cancer (Fig. 6C), suggesting that STC1 could be a biomarker for poor prognosis in HCC. The STC1 expression level was assessed by IHC staining in a total of 72 HCC tissues and matched adjacent normal liver tissues (Fig. 6D). The data indicated that HCC patients with high STC1 expression had shorter median overall survival times than patients with low STC1 expression (19 months vs. 37 months, HR = 2.30, 95% CI 1.25–4.22, **P = 0.0051) (Fig. 6E). We next examined the correlations between STC1 expression and multiple clinicopathological characteristics. Our data indicated that high STC1 expression was significantly correlated with advanced TNM stage (Table 1, *P = 0.011, χ^2^ test). Together, these findings suggest that STC1 is upregulated and correlated with poor survival in HCC patients and that it might be a biomarker for stratified analysis of HCC patient prognosis.

**Table 1:** The expression of STC1 related to clinicopathological features in HCC patients

Clinical features	High STC1 (n = 38)	Low STC1 (n = 34)	*P*-value
Age			
Young (≤ median, 52)	23	19	0.83
Old (> medium, 52)	15	15	
Gender			
Male	32	29	0.35
Female	6	5	
Overall survival, median, months	18.5 months	37 months	0.0051**
HBsAg			
Negative	10	7	0.28
Positive	28	27	
Serum AFP (ng/ml)			
Low (≤ 20)	15	12	0.32
High (> 20)	23	22	
Differentiation status			
Well differentiation	9	13	0.08
Moderately to poorly differentiation	29	21	
Tumor size			
Small (≤ 5 cm)	7	10	0.17
Large (> 5 cm)	31	24	
TNM stage^a^			
Early stage (I–II)	27	26	0.011*
Advanced stage (III–IV)	11	8	
Lymph node metastasis			
Absence	29	30	0.75
Presence	9	4	
Vascular infiltration			
Absence	30	32	0.26
Presence	8	2	

^a^AJCC/UICC T staging system

^*^p < 0.05, **p < 0.01, significant difference (χ^2^ test and Fisher’s exact test)

## Discussion

In studies aimed at understanding the roles of CAFs in HCC stemness, we uncovered evidence that CAFs could secrete STC1 and promote HCC stemness. We provided evidence that CAF-derived STC1 played a vital role in the CAF-induced promotion of HCC stemness in vitro and in vivo. Our mechanistic studies demonstrated that CAF-derived STC1 promoted HCC stemness in a Notch1-dependent manner. STC1 could activate the Notch1 signaling pathway and directly bind to the Notch1 receptors. We also revealed that STC1 was positively regulated by Notch1 at the transcriptional level in HCC cells. These studies indicated that the STC1 and the Notch1 signal formed a stromal-tumor amplifying feedforward signal to promote HCC stemness. Furthermore, STC1 was related to the poor prognosis of HCC patients and might serve as a promising biomarker for HCC patients.

CSCs can fuel and maintain tumor growth at low cell numbers and are considered to be the driving force of tumor recurrence and metastasis [3]. CAFs, one of the most important activating components of the TME, were reported to secrete a variety of secretory factors to regulate or maintain cancer stemness [38]. More recently, Song et al. found that CAFs secreted CLCF to stimulate the secretion of CXCL6 and TGF-β and then promoted cancer stemness. Our previous study also indicated that CAFs could promote the stem-like properties of HCC cells by activating HGF/IL6/STAT3 paracrine signaling pathway in vitro and in vivo [36]. Consistent with the above findings, we found CAFs enhanced the self-renewal, proliferation, resistance to sorafenib, migration, and invasion abilities of HCC cells in vitro and tumorigenicity in vivo. Our study supported the previous conclusions that CAFs could promote the stemness and malignant progression of HCC. Recent studies also reported that CAFs sometimes played the tumor-suppressing function [7]. Heterogeneity may be an important reason for CAFs to play different roles in cancer.

STC1 was a secreted protein that existed in the TME and was associated with the malignant progression of various tumors, such as breast cancer and pancreatic ductal adenocarcinoma (PDAC) [18–20]. In a study of colorectal cancer, it was found that STC1 could be secreted by CAFs and promote cancer metastasis [21]. Subsequently, studies in breast cancer and PDAC also spotted that STC1 could be secreted by CAFs [22, 23]. Additionally, evidence showed that the high expression of STC1 could promote self-renewal ability and maintain the stem-like characteristics of breast cancer stem cells. In this study, we found STC1 was more highly expressed in CAFs than that in HCC cells and NFs. CAF-derived STC1 possessed the ability to promote HCC stemness in vitro and in vivo. We respectively used STC1-Ab or lentivirus to block or inhibit STC1 of CAFs and the results confirmed that STC1 played a major role in the CAF-induced enhancement in HCC stemness.

Recent studies have shown that CAFs can regulate stemness-related pathways to maintain or promote cancer stemness [39]. The Notch signaling pathway is a highly evolutionarily conserved stemness-related pathway. In breast cancer, Notch signaling drives crosstalk between tumor cells and CAFs to promote the radio-resistance of tumor cells [40]. Here, our data indicated that CAF-derived STC1 could activate the Notch1 signaling pathway in HCC. Our previous study reported that Notch1 signaling participated in CAF-mediated HCC stemness promotion [31]; thus, here, we focused on the role of the Notch1 signaling pathway in the process of STC1-mediated HCC stemness. Our data showed that shNotch1 transduction and RO4929097 treatment significantly inhibited STC1-induced stemness in vitro and in vivo. These data intensively proved the important role of the Notch1 signaling pathway in facilitating STC1-induced stem cell-like properties in HCC cells.

Ligand binding to Notch receptors initiates the activation of Notch signaling. DSL (Delta/Seriate/LAG-2 family) proteins are considered to be typical ligands in the core Notch pathway. Recently, some new molecules have been reported as atypical ligands in the Notch signaling pathway, for example, epidermal growth factor-like domain 7 (EGFL7) [41] and microfiber-associated glycoprotein 1/2 (MAGP1/2) [42]. As a ligand molecule, STC1 can directly bind to a signaling pathway receptor molecule to activate the signal pathway [43]. Furthermore, STC1 can activate the Notch1 signaling pathway as an atypical ligand of the Notch1 receptor in glioma [37]. It attracted our attention whether there is a direct interaction between STC1 and Notch1 in HCC. We found the colocalization expression of STC1 and Notch1 in HCC cells and clinical HCC tissues. Our Co-IP results indicated that STC1 could directly interact with Notch1. Our study suggested that STC1 might be a nonspecific ligand in the Notch1 signaling pathway. CSL recruits histone deacetylases (HDACs) to inhibit the transcription of downstream genes of the Notch1 signaling pathway in the absence of NICD binding and promotes the transcription of target genes after NICD binding [44]. We found that the Su(H) motif of CSL, responsible for DNA binding, could bind to the promoter of STC1 at the site of -152/-143 bp. The data revealed that STC1 was a direct downstream transcriptional target of Notch1 signaling in HCC cells.

STC1 has been found to be a biomarker in many diseases [45–48]. In this study, we found that STC1 was highly expressed in HCC and was closely correlated with poor prognosis in HCC patients. Furthermore, we examined 59 HCC serum samples and demonstrated that a high serum concentration of STC1 correlated with advanced stages HCC. These data supported the important role of STC1 in HCC and showed that STC1 might be a promising biomarker for prognostic assessment in HCC.

Taken together, we demonstrated that CAF-derived STC1 activated the Notch1 signaling pathway of HCC cells, whereas the Notch1 signal promoted the STC1 transcriptional activity of HCC cells. STC1 and Notch1 signal formed a stromal-tumor amplifying feedforward signal in the TME, promoted HCC stemness, and provided new therapeutic targets for targeting both cancer cells and CAFs. We believe that more therapies targeting both cancer cells and CAFs could be developed for cancers and that these approaches could have a strategic advantage over those targeting only tumor cells or CAFs. Our study still has some limitations. In subsequent studies, there is still an urgent need to explore biosafety considerations and effective inhibitors or drugs targeting STC1/Notch1 to combat HCC. We should not ignore the role of other stemness-related pathways in CAFs and HCC. Small sample size may lead to inaccurate results, so the sample size of clinical samples needs to be further expanded. Further investigation needs to be carried out from two perspectives (the influence of CAFs on tumor cells and the influence of tumor cells on CAFs) to develop new and effective tumor treatment targets. Comprehensive exploration of the function of STC1 on tumor immune cells and the immune microenvironment will facilitate the targeted therapy of STC1 and may improve the efficacy of immunotherapy. In addition, in future research, we will further explore the crosstalk between tumor cells and tumor microenvironment components by simulating the internal environment through the establishment of the organoids.

## Conclusions

In summary, we found that CAF-secreted STC1 promoted HCC stemness and STC1 expression was positively related to Notch1 expression, poor prognosis, and advanced tumor stage in HCC. Mechanically, STC1 and Notch1 signaling formed a positive feedback loop to promote HCC stemness. And this stromal-tumor amplifying STC1-Notch1 feedforward signal could constitute an effective therapeutic target for HCC patients.

## Supplementary Information

**Additional file 1: Figure S1.** Supplemental data showing that CAF-secreted STC1 promoted the stem-like properties of HCC cells. A. A CCK8 toxicity assay was used to evaluate the viability of the indicated SNU-398 cells. B. Representative images of transwell migration and invasion assays and the histogram in the indicated SNU-398 cells. Scale bar, 100 μm. C and D. GSEA of the HCC samples in the TCGA database were performed. E. The results of cell cycle analysis of SNU-398 cells by flow cytometry. F. The results of cell apoptosis with flow cytometry. G. After treatment with sorafenib (5 μM), flow cytometry was used to analyze apoptosis of SNU-398 cells. H. The bar graph of STC1 expression in CAFs. I. The histogram analysis of colony formation in indicated cells. J. Representative images of transwell migration and invasion assays of SNU-398 cells. Scale bar, 100 μm. For the statistical analysis, ns, no significance, *P < 0.05, ** P < 0.01, and *** P < 0.001, t test.

**Additional file 2: Figure S2.** Supplemental data showing that Notch1 knocked down inhibited CAF-derived STC1-induced stemness. A. and B. Western blotting was performed to measure the protein levels of SNU-398. C. and D. The selection of shRNA sequences and the regulation of Notch1 in HCC cells. E. Viability of the indicated SNU-398 cells. F. Representative images of colony formation in different groups of HCC cells. G and H. Representative images of transwell migration and invasion assays of the indicated MHCC-97H (G) and SNU-398 (H) cells. Scale bar, 100 μm. For the statistical analysis, ns, no significance, *P < 0.05, ** P < 0.01, and *** P < 0.001, t test.

**Additional file 3: Figure S3.** Supplemental data showing that RO4929097 blocked CAF-derived STC1-induced stemness. A. The protein levels of Notch1 signaling pathway molecules were detected by western blotting. B. Representative images of the sphere formation assay in indicated cells. Scale bar, 50 μm. C. Viability of the indicated HCC cells. D. The results of colony formation assay in different groups of HCC cells. E. The migration and invasion abilities of MHCC-97H and SNU-398 cells. Scale bar, 100 μm. F. Measurement of the expression levels of NANOG, OCT4, and SOX2. For the statistical analysis, *P < 0.05, ** P < 0.01, and *** P < 0.001, t test.

**Additional file 4.** Supplemental data showing the sequence of primer and virus.

## Data Availability

Data related to this paper may be requested from the corresponding author.

## Abbreviations

CAFs: Cancer associated fibroblasts
TME: Tumor microenvironment
STC1: Stanniocalcin-1
HCC: Hepatocellular carcinoma
CoIP: Co-immunoprecipitation
NICD: Notch intracellular domain
IHC: Immunohistochemical
NFs: Normal fibroblasts
IF: Immunofluorescence
α-SMA: α-Smooth muscle actin
