# Supplementary figures and images for: The stromal-tumor amplifying STC1-Notch1 feedforward signal promotes the stemness of hepatocellular carcinoma
Source: J Transl Med. 2023 Mar 31;21:236. doi: 10.1186/s12967-023-04085-8 (PMC10067215; doi:10.1186/s12967-023-04085-8)

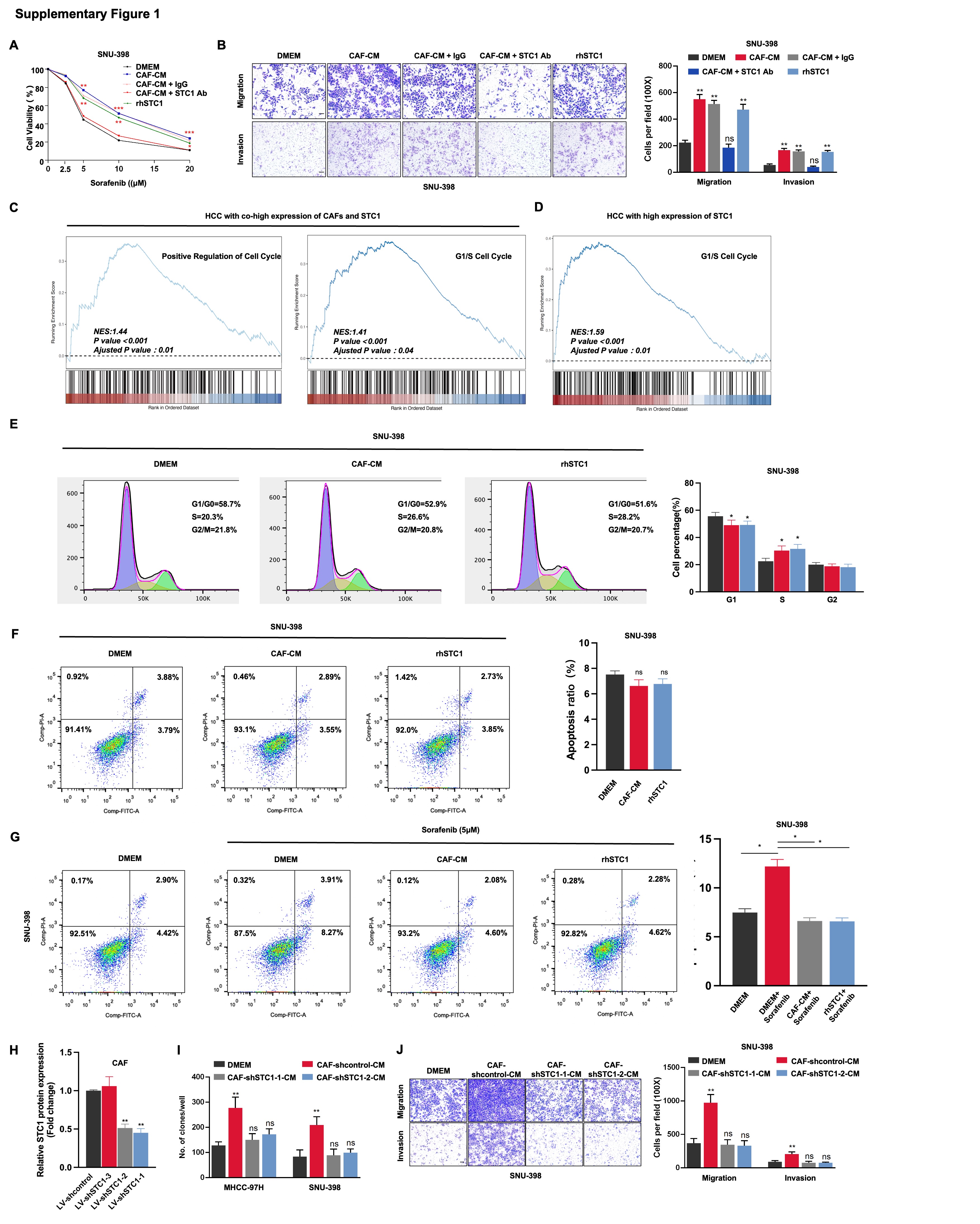

Supplement: Supplementary file 1 — Additional file 1: Figure S1. Supplemental data showing that CAF-secreted STC1 promoted the stem-like properties of HCC cells. A. A CCK8 toxicity assay was used to evaluate the viability of the indicated SNU-398 cells. B. Representative images of transwell migration and invasion assays and the histogram in the indicated SNU-398 cells. Scale bar, 100 μm. C and D. GSEA of the HCC samples in the TCGA database were performed. E. The results of cell cycle analysis of SNU-398 cells by flow cytometry. F. The results of cell apoptosis with flow cytometry. G. After treatment with sorafenib (5 μM), flow cytometry was used to analyze apoptosis of SNU-398 cells. H. The bar graph of STC1 expression in CAFs. I. The histogram analysis of colony formation in indicated cells. J. Representative images of transwell migration and invasion assays of SNU-398 cells. Scale bar, 100 μm. For the statistical analysis, ns, no significance, *P < 0.05, ** P < 0.01, and *** P < 0.001, t test. [file 12967_2023_4085_MOESM1_ESM.jpg]

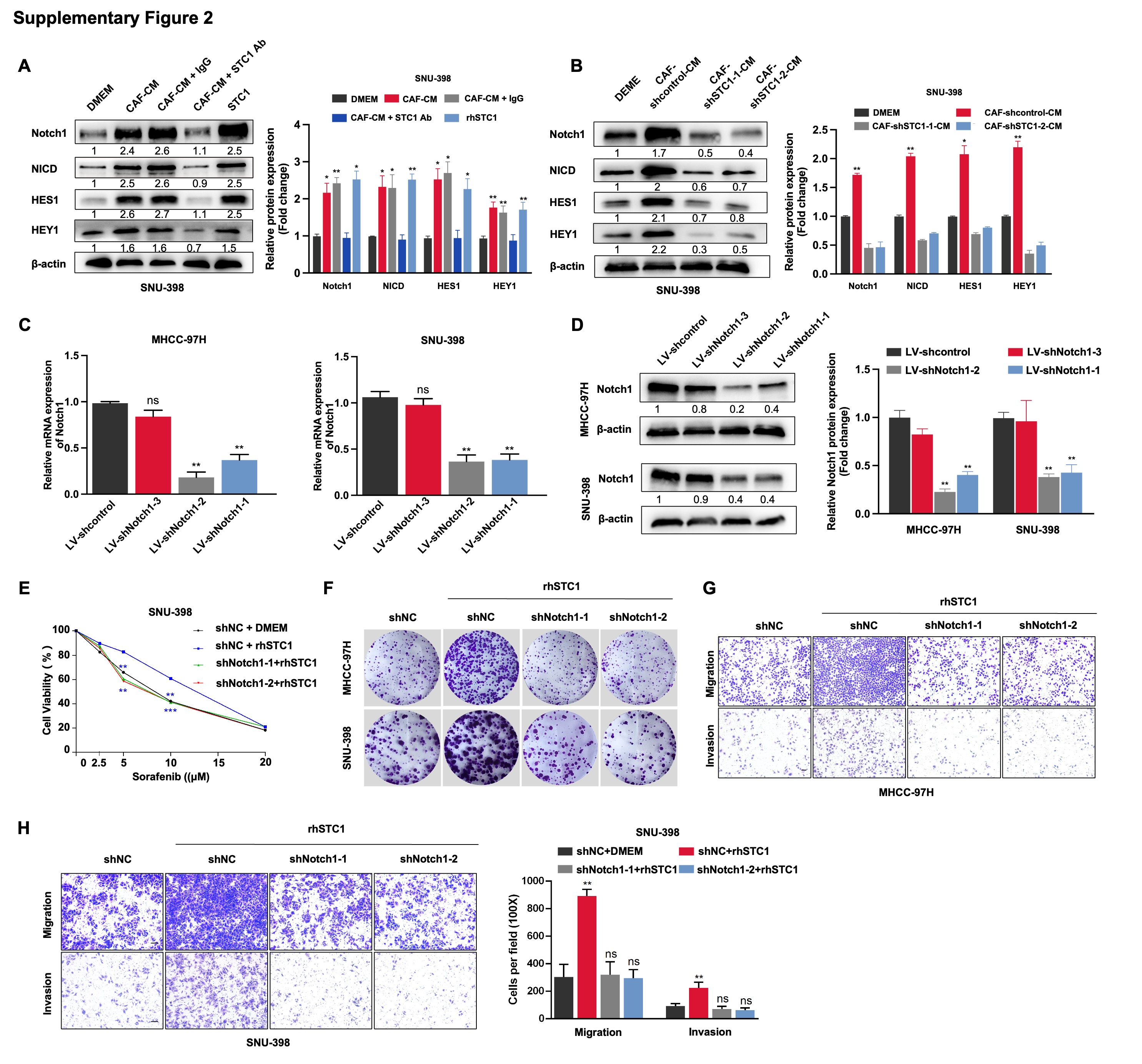

Supplement: Supplementary file 2 — Additional file 2: Figure S2. Supplemental data showing that Notch1 knocked down inhibited CAF-derived STC1-induced stemness. A. and B. Western blotting was performed to measure the protein levels of SNU-398. C. and D. The selection of shRNA sequences and the regulation of Notch1 in HCC cells. E. Viability of the indicated SNU-398 cells. F. Representative images of colony formation in different groups of HCC cells. G and H. Representative images of transwell migration and invasion assays of the indicated MHCC-97H (G) and SNU-398 (H) cells. Scale bar, 100 μm. For the statistical analysis, ns, no significance, *P < 0.05, ** P < 0.01, and *** P < 0.001, t test. [file 12967_2023_4085_MOESM2_ESM.jpg]

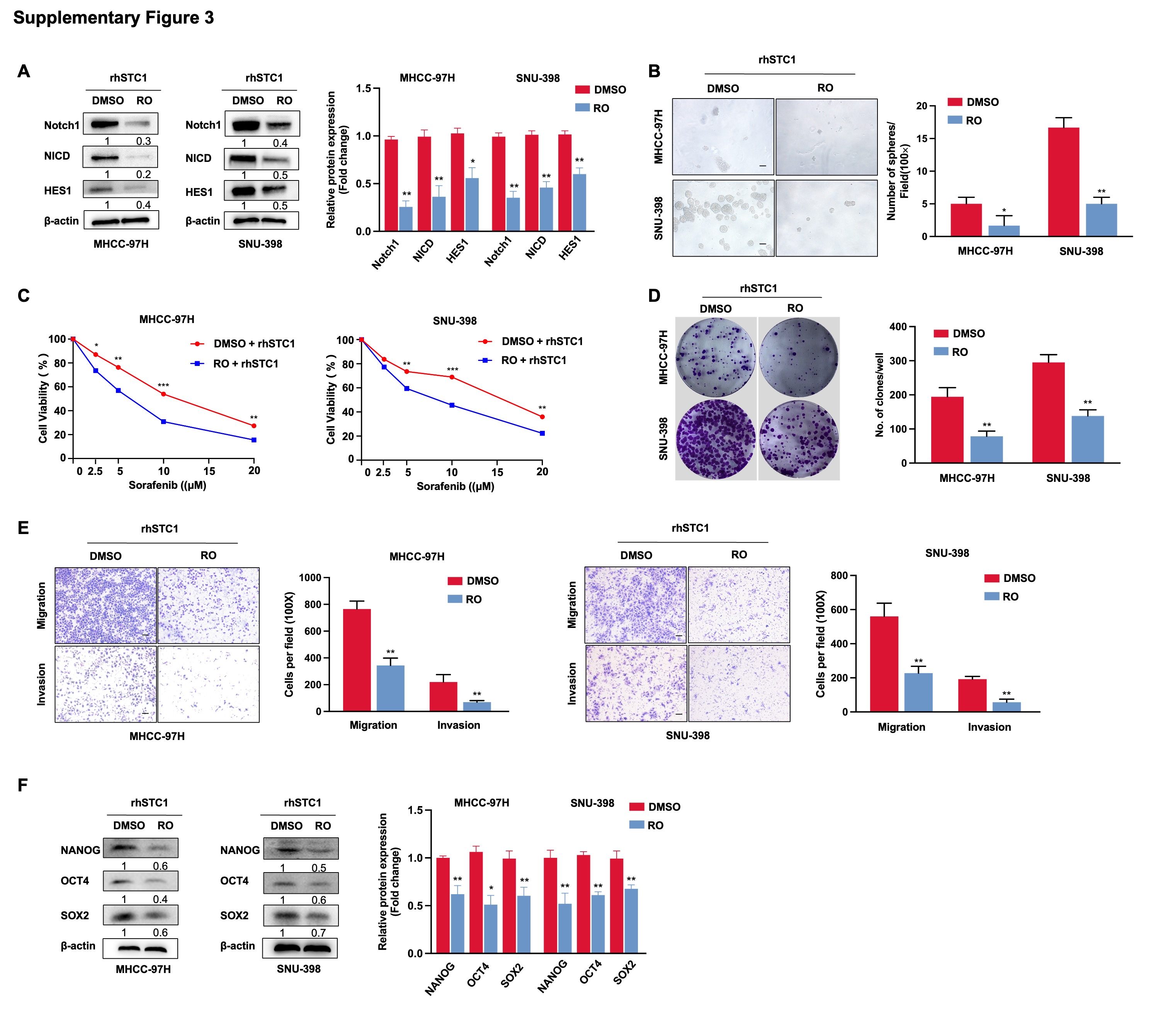

Supplement: Supplementary file 3 — Additional file 3: Figure S3. Supplemental data showing that RO4929097 blocked CAF-derived STC1-induced stemness. A. The protein levels of Notch1 signaling pathway molecules were detected by western blotting. B. Representative images of the sphere formation assay in indicated cells. Scale bar, 50 μm. C. Viability of the indicated HCC cells. D. The results of colony formation assay in different groups of HCC cells. E. The migration and invasion abilities of MHCC-97H and SNU-398 cells. Scale bar, 100 μm. F. Measurement of the expression levels of NANOG, OCT4, and SOX2. For the statistical analysis, *P < 0.05, ** P < 0.01, and *** P < 0.001, t test. [file 12967_2023_4085_MOESM3_ESM.jpg]
