# Supplementary material for: The stromal-tumor amplifying STC1-Notch1 feedforward signal promotes the stemness of hepatocellular carcinoma
Source: J Transl Med. 2023 Mar 31;21:236. doi: 10.1186/s12967-023-04085-8 (PMC10067215; doi:10.1186/s12967-023-04085-8)
Supplement: Supplementary file 4 — Additional file 4. Supplemental data showing the sequence of primer and virus. [file 12967_2023_4085_MOESM4_ESM.docx]

**Table S1.** The sequence of primer and virus

| **Primer or virus** | **sequence** |
| --- | --- |
| Nocth1 primer | Forward primer: TGGACCAGATTGGGGAGTTC |
|  | Reverse primer: GCACACTCGTCTGTGTTGAC |
| STC1 primer | Forward primer: AGGTGCAGGAAGAGTGCTACA |
|  | Reverse primer: GACGACCTCAGTGATGGCTT |
| β-actin primer | Forward primer: CATGTACGTTGCTATCCAGGC |
|  | Reverse primer: CTCCTTAATGTCACGCACGAT |
| Notch1 shRNA | shNotch1-1, 5′-GGAGCATGTGTAACATCAA-3′ |
|  | shNotch1-2, 5’- TGCCAACATCCAGGACAACAT -3’ |
|  | shNotch1-3, 5’- AGGGAGCATGTGTAACATCAA -3’ |
| STC1 shRNA | shSTC1-1, 5’-GCATTCGTCAAAGAGAGCTTA-3’ |
|  | shSTC1-2, 5’-GCCCAATCACTTCTCCAACAG-3’ |
|  | shSTC1-3, 5’- GCACAATCAGAGACAGCCTGA-3’ |
